# Supplementary figures and images for: Novel machine learning method allerStat identifies statistically significant allergen-specific patterns in protein sequences
Source: J Biol Chem. 2023 Apr 21;299(6):104733. doi: 10.1016/j.jbc.2023.104733 (PMC10209033; doi:10.1016/j.jbc.2023.104733)

Fig. S1 (1)

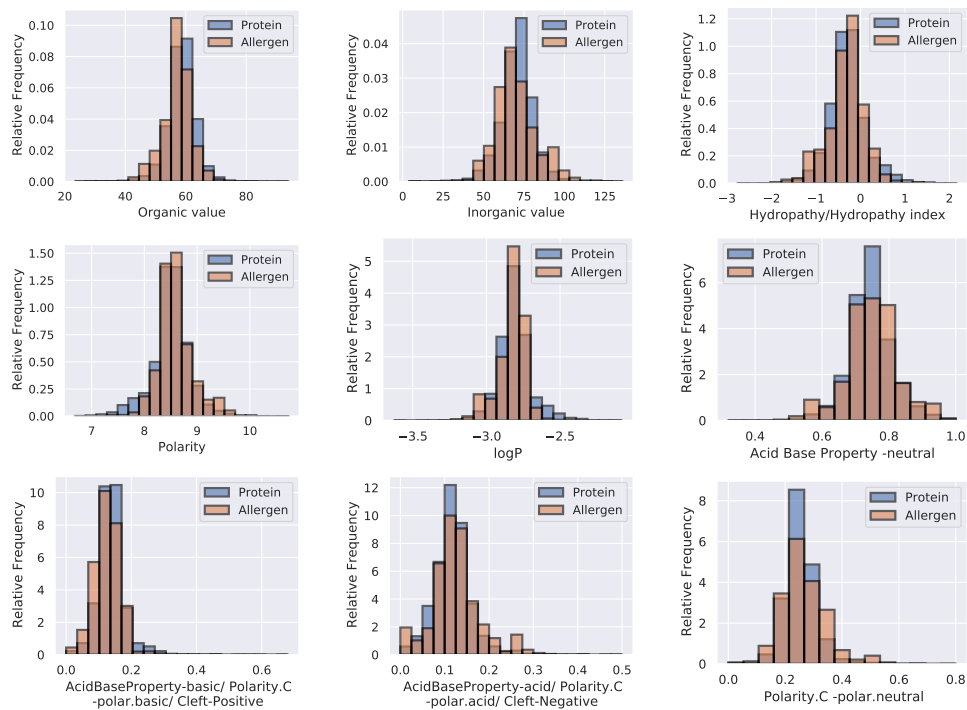

Fig. S1 (2)

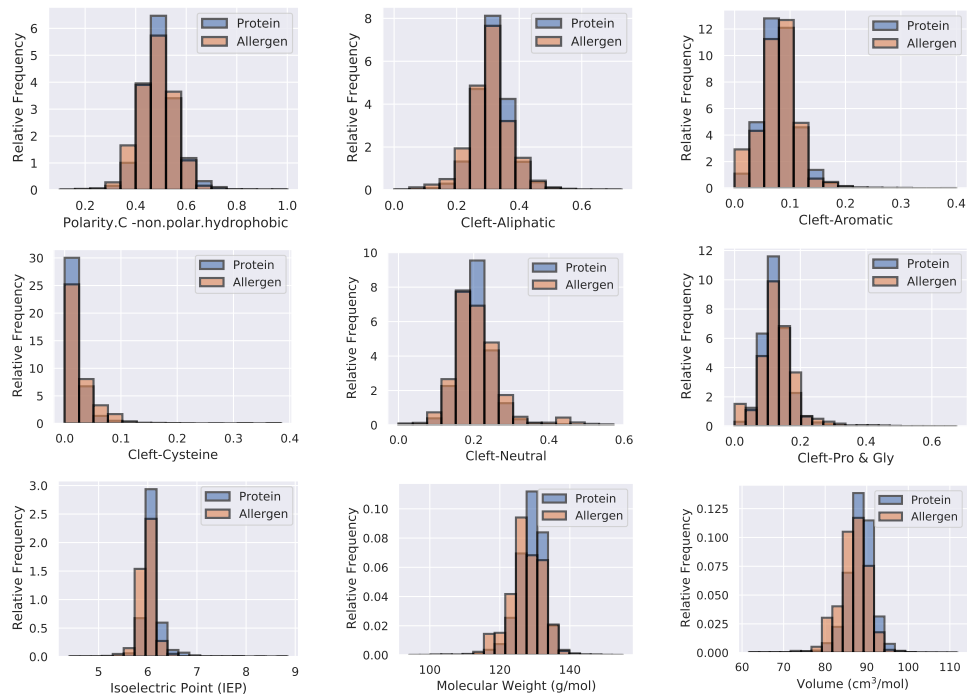

Supplement: supporting-figure1 [file mmc11.pdf]

Fig. S2

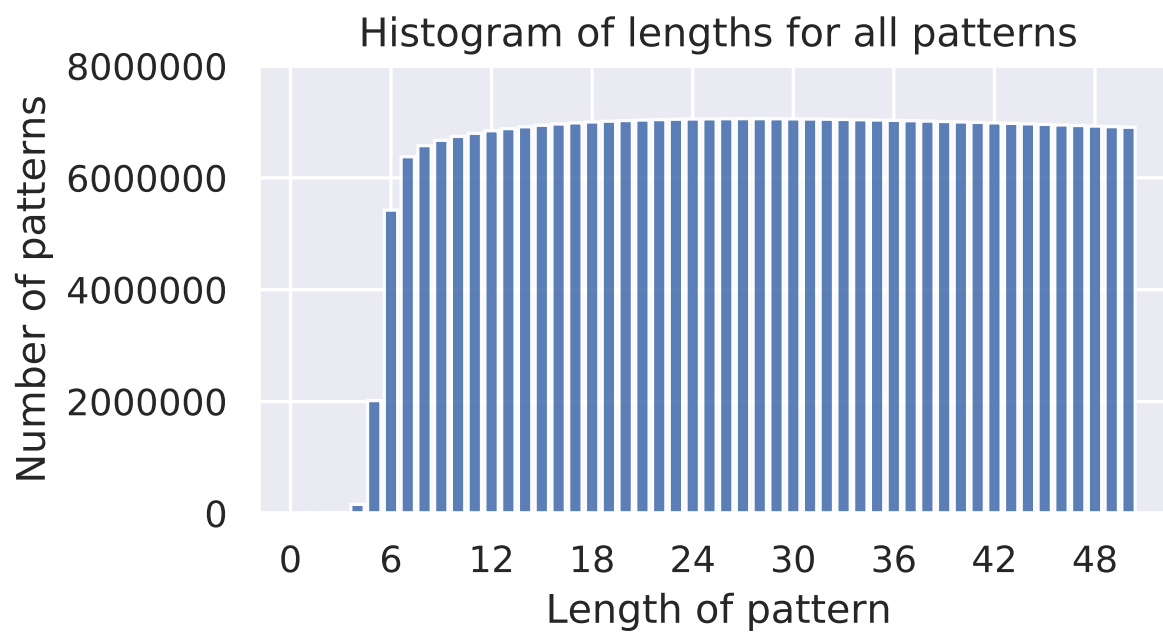

Supplement: supporting-figure2 [file mmc12.pdf]
