# Supplementary material for: Novel machine learning method allerStat identifies statistically significant allergen-specific patterns in protein sequences
Source: J Biol Chem. 2023 Apr 21;299(6):104733. doi: 10.1016/j.jbc.2023.104733 (PMC10209033; doi:10.1016/j.jbc.2023.104733)
Supplement: supporting-figure3 [file mmc13.pdf]

Fig. S3

Bovine and Goat

```
Pattern  538    1 QQQPPFSQQQPILPQQPPFSQQQQLVLPQQSPFSQQQQ--------
Bos t           1 QSEEQQQTEDELQDKIHPFAQTQSLVYPFPGP--------------
Car h           1 ---------------IHPFAQAQSLVYPFTGPIPNSLPQNILPLTQ
```

Yorkshire-fog and Crayfish

```
Pattern  614    1 -------------TACGNVPIFKDGKGCGSCYE----
Hol l 1         1 GYKDVDKPPFSGMTGCGNTPIFKDGRGCGSCFEIK--
Pro c           1 ----------------------FKDRKDGSCYVSYKV
```

Chicken and Cedar

```
Pattern  688    1 ALGDTLEKICNEIKIVATPDGGC---------
Gal d 1         1 ----------------KRHDGGCRKE------
Jun a 3         1 ------------------VDGGCNSACNVFKT
```

Melon and Sunflower and Crayfish

```
Pattern  718    1 VKKTGQALIFGIYDEPVTPGQCNMIVERLGDYLVEQGM
Cuc m 2         1 ------------YDEPLTPGQCNMIVE-----------
Pro c           1 ---------RIIYGGSVTPGNCKE--------------
Hel a 2         1 ------------YDEPVAPG------------------
```

Soybean and Bovine

```
Pattern  856    1 NQLDQFPRRFYLAGNQEQEFLRYQQQ----
Gly m           1 --------RFYLAGNQEQEFLKY-------
Bos t           1 ----------PQRDMPIQAFLLYQEPVLGP
```

Cladosporium and Penicillum and Pea

```
Pattern  885    1 ---------------------LSGTSMASPHIAGLL---------------------
Cla h 9         1 IFAPGQDILSAWIGSTTATNTISGTSMATPHIVGLSVYLMGLENLSGPAAVTARIKE
Pen ch 18       1 -------ILSTWVGSDHATNTISGTSMA-----------------------------
Pis s           1 --------AAHEVLSWSFHSELSGTSSSKQ---------------------------
```
